# Limb asymmetry in swimmers and its correlation with sprint performance

**DOI:** 10.3389/fphys.2026.1809652

**Published:** 2026-04-22

**Authors:** Jinjin Dai, Junjun Xu, Jianjun Lin

**Affiliations:** 1 Department of Sports, Zhejiang Wanli University, Ningbo, China; 2 School of Information Technology and Artificial Intelligence, Zhejiang University of Finance and Economics, Hangzhou, China; 3 Faculty of Physical Education, Ningbo University, Ningbo, China

**Keywords:** inter-limb asymmetry, isokinetic strength, sex differences, sprint swimming, swimming performance

## Abstract

**Background:**

Inter-limb strength asymmetry is well-established as an injury risk factor, yet its relationship with athletic performance remains controversial and context-dependent. In swimming-a bilateral cyclical sport theoretically requiring symmetrical force production-the characteristics and performance implications of strength asymmetry remain insufficiently understood, particularly regarding contraction velocity and sex.

**Objective:**

This study aimed to: (1) characterize shoulder and hip isokinetic strength asymmetry in competitive swimmers at 60°/s and 180°/s; (2) explore sex differences in asymmetry profiles; and (3) examine associations of absolute strength and strength asymmetry with sprint performance.

**Methods:**

Thirty-two swimmers (20 males, 12 females) aged 18–22 years completed isokinetic assessments of shoulder flexion/extension, shoulder internal/external rotation, and hip flexion/extension at 60°/s and 180°/s. Asymmetry indices were calculated using the percentage difference method. Swimming performance was evaluated via 15-m start, turn, and 100-m freestyle times. Between-session reliability was established (ICC = 0.988–0.998; CV = 1.85%–6.25%). Mann–Whitney U tests, Wilcoxon signed-rank tests, and Spearman rank correlation were performed.

**Results:**

Males demonstrated significantly greater absolute strength and faster performance times than females (all p<0.001). Mann–Whitney U tests revealed a significant sex difference in hip extension asymmetry at 180°/s, with female swimmers exhibiting greater asymmetry than males (p = 0.019). Asymmetry indices were generally higher at 180°/s than at 60°/s, with significant velocity-dependent increases only for shoulder external rotation in males (Z = –2.373, p = 0.016, r = 0.531). Spearman rank correlation showed that, in females, hip extension torque at 60°/s was significantly positively correlated with turn time (left: rho = 0.679, p < 0.05; right: rho = 0.618, p < 0.05). In males, left shoulder extension at 60°/s and 180°/s showed significant negative correlations with turn time (rho = –0.470 and –0.531, respectively, both p < 0.05). No significant correlations were observed between any asymmetry index and performance in either sex.

**Conclusion:**

Strength asymmetry in swimmers is movement-, velocity-, and sex-specific, with female swimmers exhibiting greater hip extension asymmetry at high velocity. Absolute hip extensor strength is significantly associated with turn performance in females, whereas in males, shoulder extensor strength correlates negatively with turn time. Strength asymmetry is generally unrelated to swimming performance, supporting the view that asymmetry should not be universally deemed detrimental. These findings provide evidence-based guidance for individualized strength diagnostics in competitive swimming.

## Introduction

In competitive sports, the pursuit of optimal performance necessitates a thorough understanding of both enhancing factors and potential risk factors. Inter-limb asymmetry, defined as a measurable difference in function, strength, or morphology between contralateral limbs, has garnered significant attention in sports science. Substantial evidence confirms that pronounced strength asymmetries elevate the risk of musculoskeletal injuries (Croisier et al., 2008; Brumitt et al., 2013). However, the impact of asymmetry on athletic performance remains a subject of considerable debate (Bishop et al., 2022).

The prevailing “balanced development” paradigm suggests that asymmetry can impair foundational physical capacities such as jumping, sprinting, and change-of-direction ability (Bell et al., 2014; Bishop et al., 2018, 2021; Michailidis et al., 2020). Conversely, evidence from sports like soccer, sprinting, and combat sports indicates that asymmetry is not only prevalent among elite athletes but may also represent a sport-specific, non-detrimental adaptation (Schiltz et al., 2009; Buckeridge et al., 2012; Stanton et al., 2015; Exell et al., 2017; Ferreira et al., 2018). This contradiction underscores that the functional implications of asymmetry are likely context-dependent, modulated by sport-specific biomechanics and athlete expertise.

Swimming, characterized by bilateral, alternating, and cyclical movements, presents a unique and critical context for this debate. Theoretically, symmetrical limb contribution is essential for maintaining body alignment, minimizing drag, and ensuring efficient propulsion (Sanders et al., 2015; Barbosa et al., 2010). Consequently, significant asymmetry might be deemed detrimental to performance (dos Santos et al., 2013). Yet, empirical findings are inconsistent. While some studies report minimal asymmetries in swimmers (Psycharakis et al., 2021; Carvalho et al., 2019), others document notable inter-limb differences in muscle power, range of motion, and in-water force production (Potts et al., 2002; Pereira et al., 2019; Morouço et al., 2015). Potential sources of asymmetry in swimmers include one-sided breathing patterns, technical biases, training habits, and injury history (Maloney, 2019; Seifert et al., 2005).

This inconsistency in the literature is compounded by methodological diversity in asymmetry assessment—encompassing various dry-land and in-water tests, and different calculation and interpretation thresholds (Knihs et al., 2022). Crucially, many studies fail to distinguish “real” asymmetry from measurement noise by comparing inter-limb differences to the inherent variability (test reliability) of the assessment tool—a recommended practice in asymmetry research (Bishop et al., 2021). This methodological shortcoming obscures a clear understanding of asymmetry’s true prevalence and relevance in swimming.

Furthermore, critical knowledge gaps persist. First, asymmetry is task-specific (Bishop et al., 2018), yet its manifestation across different strength qualities (e.g., maximal strength at slow velocities vs. power at high velocities) in swimmers is understudied. Second, while physiological and training adaptations can differ between sexes, whether and how asymmetry profiles manifest differently in male and female swimmers remains largely unexamined. Any such analysis in the current study will be considered exploratory, given the sample size considerations, but it represents a necessary first step to inform future, adequately powered investigations. Finally, the relative importance of absolute strength levels versus symmetry in determining key components of sprint swimming performance (e.g., start, turn, and overall race time) is unknown.

Therefore, this study aims to address these gaps by systematically investigating: 1) the characteristics of shoulder and hip strength asymmetry in competitive swimmers at different contraction velocities; 2) preliminarily exploring potential differences in these asymmetry profiles between male and female swimmers, with the acknowledgment that this aspect is exploratory due to the sample composition; and 3) the association between both absolute strength and asymmetry metrics with sprint-specific performance outcomes.

## Methods

### Participants

A total of 32 competitive swimmers (20 male, 12 female; age: 19.44 ± 1.44 years; aged 18–22 years; height: 177.77 ± 6.84 cm; body mass: 71.88 ± 8.50 kg; body mass index: 22.68 ± 1.67 kg/m²) from Beijing Sport University participated in this study. According to the Chinese Swimming Association classification system, 9 participants were classified as National-level athletes, while the remaining 23 were Class I athletes. All participants were actively competing at the national university level. The basic information differences existed between sexes as detailed in **Table 1**. All participants confirmed their lack of significant injuries in the preceding six months and provided written informed consent after a comprehensive explanation of the study’s aims and methods. Swimmers usually perform 3–4 swim drills per week and at least 2 structured strength and conditioning sessions per week during the testing cycle. All subjects provided written informed consent and their personal information was handled anonymously. For female participants, only those who were not in the menstrual phase at the time of testing were included. Initially, 14 female swimmers were recruited; two were excluded due to their menstrual cycle status, resulting in a final sample of 12 female participants. The research protocol was approved by the Ethics Committee of Beijing Sport University (2023208H).

**Table 1:** Baseline data of the subjects.

Basic information	Total (n=32)	Male (n=20)	Female (n=12)
Age (years)	19.44 ± 1.44	19.40 ± 1.67	19.50 ± 1.00
Height (cm)	177.77 ± 6.84	181.11 ± 5.06	172.17 ± 5.84
Body mass (kg)	71.88 ± 8.50	76.06 ± 7.2	65.59± 7.62
Body mass index (kg/m²)	22.68± 1.67	23.19 ± 1.77	22.04 ± 1.51
Training Time (years)	10.27± 2.48	9.43± 2.31	11.75± 2.09
Sprint (50-100m)	24 (75%)	15 (75%)	9 (75%)
Mid-distance (200-400m)	5 (15.6%)	3 (15%)	2 (16.7%)
Long-distance (800-1500m)	3 (9.4%)	2 (10%)	1 (8.3%)

### Experimental design

This study recruited swimmers from Beijing Sport University. Conducted in September 2023, the protocol involved isokinetic muscle strength testing (60–80 min) and 100 m sprint testing (20–30 min) on separate days to mitigate fatigue effects. All testing sessions were scheduled based on participants’ availability and recovery status. Isokinetic strength testing was conducted on weekdays (Monday to Friday), while swimming performance testing was performed on Saturday. To minimize the influence of confounding factors, participants were instructed to: (1) refrain from consuming caffeine, alcohol, or any medications within 24 hours prior to each testing session; (2) report any signs of infection, injury, or perceived fatigue on the testing day; and (3) maintain their regular training routine but avoid strenuous training within 48 hours before testing. Participants who reported any illness, acute injury, or excessive fatigue were rescheduled.

Participants were acquainted with testing procedures, and their information was documented according to study protocols. To ensure data validity and reliability, subjects completed a warm-up and were advised to avoid strenuous physical activity for 48 hours before testing and to fast for 2 hours beforehand.

### Procedures

#### Isokinetic strength testing

**Figure 1:**
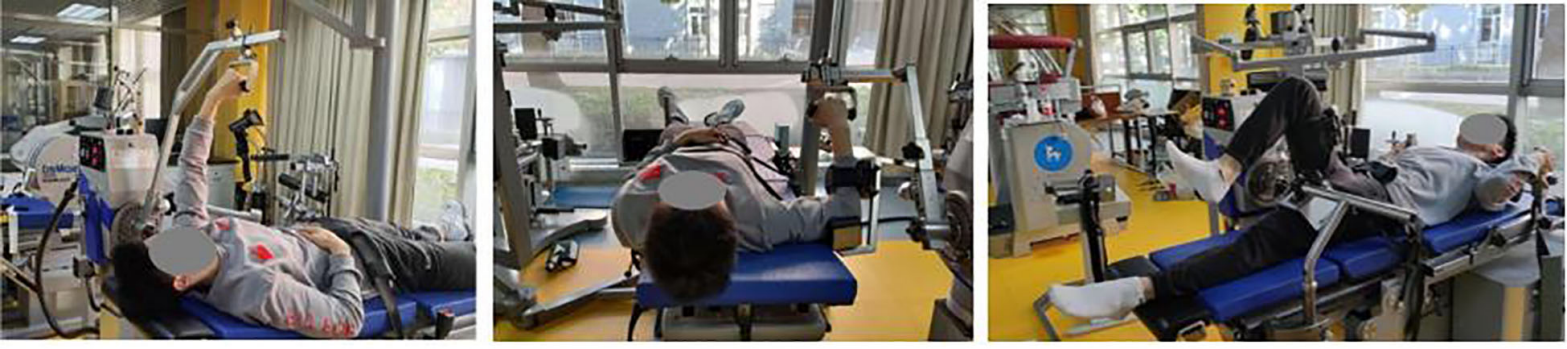
Schematic diagram of isokinetic testing.

Isokinetic strength testing (**Figure 1**) was conducted using the Isomed 2000 dynamometer (D&R Ferstl GmbH, Hemau, Germany). Test selections were grounded in the biomechanical demands of front-crawl swimming: shoulder internal/external rotation was assessed to evaluate glenohumeral stability and pull-path control, shoulder flexion/extension to reflect propulsion-generating capacity during the pull phase, and hip flexion/extension to represent leg-kick propulsion and push-off power during turns and starts. Two angular velocities were employed for each joint action—60°/s to represent maximal strength and 180°/s to represent speed-strength—both of which are considered performance-determining qualities in sprint swimming (Boettcher et al., 2020).

#### Shoulder protocol

Participants were positioned supine with the shoulder abducted to 90° and the elbow flexed to 90°, consistent with manufacturer guidelines for isolated glenohumeral assessment. Stabilization was achieved via straps securing the torso, chest, and abdomen. The range of motion for internal/external rotation was set from 90° external rotation to 90° internal rotation; for flexion/extension, the range spanned from -5° to +170°. Testing commenced with the dominant limb at 180°/s (5 repetitions), followed by a 3-minute rest, then proceeded at 60°/s (5 repetitions). The non-dominant limb was tested thereafter following an identical sequence. Each contraction was performed through a full, active range of motion. Participants completed 3–5 submaximal familiarization trials prior to each velocity condition. Standardized verbal encouragement was provided throughout; visual feedback was withheld. Peak torque (Nm/kg) was automatically identified by the dynamometer’s software as the highest value from five maximal repetitions and normalized to body mass.

#### Hip protocol

Hip flexion and extension strength were assessed isokinetically at identical velocities (60°/s and 180°/s). For flexion, participants lay supine with the hip and knee stabilized; for extension, a prone position was adopted. Range of motion was set from 10° to 100° of hip flexion. Limb order and velocity progression followed the shoulder protocol: 180°/s first (5 repetitions), rest, then 60°/s (5 repetitions). Identical familiarization, stabilization, and verbal encouragement procedures were applied. Peak torque (Nm/kg) served as the primary outcome variable.

All testing was conducted by the same certified investigator at consistent times of day (± 1 hour) to control for circadian variation. Individual dynamometer settings (seat position, pad adjustments, lever arm length) were recorded during the first session and reproduced exactly across all subsequent tests. A standardized 15-minute warm-up—comprising dynamic stretching, limb activation, pillar preparation, and medicine ball throws—was completed prior to each assessment. Total testing duration per session ranged from 60 to 80 minutes. Gravity correction was not applied, in accordance with the manufacturer’s recommendation for this testing position.

#### Sports performance testing

The swimming performance tests were conducted in a 50-meter indoor pool maintained at 28 °C. The pool lanes were marked with 50-meter spiral float line ropes. Two high-speed waterproof cameras (GoPro HERO7, GoPro Inc, San Mateo, CA, USA) recording at 2.7K and 100Hz were utilized: one positioned 0.15 m underwater at the lane center for stroke parameter analysis (Franken et al., 2013), and another mobile camera operated by a researcher to capture the entire trial. Sprinting time was measured by a coach using a chronomete (Franken et al., 2013; Khiyami et al., 2022). Prior to the trial, swimmers performed 15 minutes of warm-up exercises. The performance metrics measured were the total 100 m time, as well as split times at 15 m (start), the turn (5 m pre-turn to 15 m post-turn).

### Statistical analyses

The data are presented as means with standard deviations (SD). Statistical analyses were conducted using IBM SPSS Statistics (version 27; SPSS, Inc., Armonk, NY, USA). Normality was assessed using the Shapiro–Wilk test, and homogeneity of variances was assessed using Levene’s test. Given the relatively small sample size (particularly for female subgroups, n = 12) and the heterogeneity of the sample, some variables violated the assumptions required for parametric tests. Accordingly, non-parametric tests were employed for all inferential analyses to ensure robustness.

All isokinetic strength data were obtained through three sets of tests, each consisting of five maximal voluntary isokinetic contractions. Sufficient rest was provided between sets to eliminate fatigue. The highest peak torque value from each set was recorded as the representative value for that set. Absolute and relative reliability were calculated using the coefficient of variation (CV) and intraclass correlation coefficient (ICC) with absolute agreement (95% confidence intervals), respectively. CV values < 10% were considered acceptable (Cormack et al., 2008) and ICC values were interpreted according to the guidelines proposed by Koo and Li (2016), where > 0.9 = excellent, 0.75 to 0.9 = good, 0.5 to 0.74 = moderate, and < 0.5 = poor.

Interlimb asymmetries were quantified using a standard percentage difference equation: 100/(max value) * (min value)*-1 + 100 (Bishop et al., 2021; Cuthbert et al., 2021). The Wilcoxon signed-rank test was used to examine differences in asymmetry values between different angular velocities (60°/s vs. 180°/s). The Mann–Whitney U test was used to compare asymmetry indicators between male and female swimmers. Spearman’s rank correlation was used to examine relationships between limb strength measures and swimming performance. Effect sizes (r) were calculated for all non-parametric tests and interpreted as small (0.1), medium (0.3), or large (0.5) (Cohen, 1988). For correlation magnitudes, the following criteria were applied: 0.00 to 0.09 = trivial, 0.10 to 0.29 = small, 0.30 to 0.49 = moderate, 0.50 to 0.69 = large, 0.70 to 0.89 = very large, 0.90 to 0.99 = nearly perfect, and 1.00 = perfect (Hopkins et al., 2009). Statistical significance was set at p < 0.05.

## Result

As shown in **Table 2**, male swimmers demonstrated significantly higher peak torque values than female swimmers in all 24 shoulder and hip isokinetic strength assessments (all p<0.001). Male swimmers also recorded significantly faster times than female swimmers in the 15-m start, turn phase, and overall 100-m performance (all p<0.001).

**Table 2:** Mean testing data ± standard deviations for each group.

Test	Total (n =32)	Male (n=20)	Female (n=12)	*P* (sex)
S-LF 60°/s PT (Nm)	50.41 ± 12.97	57.72 ± 10.11	38.45 ± 6.64	< 0.001
S-LE 60°/s PT (Nm)	79.79 ± 22.20	92.39 ± 16.06	59.18 ± 13.71	< 0.001
S-RF 60°/s PT (Nm)	47.03 ± 12.21	54.17 ± 9.53	35.36 ± 4.65	< 0.001
S-RE 60°/s PT (Nm)	80.21 ± 22.12	92.11 ± 17.63	60.73 ± 13.02	< 0.001
S-LF180°/s PT (Nm)	48.07 ± 14.31	55.83 ± 9.91	35.36 ± 10.96	< 0.001
S-LE 180°/s PT (Nm)	78.76 ± 24.89	91.67 ± 19.05	57.64 ± 18.11	< 0.001
S-RF180°/s PT (Nm)	46.27 ± 14.07	53.94 ± 11.56	33.73 ± 6.94	< 0.001
S-RE 180°/s PT (Nm)	83.59 ± 23.82	97.33 ± 17.72*	61.09 ± 12.58	< 0.001
S-LIR 60°/s PT (Nm)	44.41 ± 12.95	50.89 ± 11.58	33.82 ± 6.54	< 0.001
S-LER 60°/s PT (Nm)	28.59 ± 7.01	32.44 ± 5.29	22.27 ± 4.41	< 0.001
S-RIR 60°/s PT (Nm)	47.76 ± 14.00	55.5 ± 11.33*	35.09 ± 6.79	< 0.001
S-RER 60°/s PT (Nm)	30.86 ± 7.63	35.00 ± 5.91*	24.09 ± 4.72	< 0.001
S-LIR180°/s PT (Nm)	47.59 ± 15.71	55.67 ± 13.84	34.36 ± 7.43	< 0.001
S-LER 180°/s PT (Nm)	24.55 ± 8.05	28.78 ± 7.15	17.64 ± 3.11	< 0.001
S-RIR180°/s PT (Nm)	47.93 ± 14.58	55.94 ± 11.53	34.82 ± 8.02	< 0.001
S-RER 180°/s PT (Nm)	28.31 ± 10.18	33.56 ± 9.35	19.73 ± 3.35	< 0.001
H-LF 60°/s PT (Nm)	107.17 ± 24.20	119.78 ± 18.74	86.55 ± 17.00	< 0.001
H-LE 60°/s PT (Nm)	267.17 ± 59.47	295.89 ± 51.92	220.18 ± 37.5	< 0.001
H-RF 60°/s PT (Nm)	109.62 ± 30.39	125.83 ± 25.93	83.09 ± 14.03	< 0.001
H-RE 60°/s PT (Nm)	272.24 ± 74.78	312.39 ± 62.7*	206.55 ± 36.39	< 0.001
H-LF180°/s PT (Nm)	104.72 ± 30.55	116.72 ± 30.15	85.09 ± 19.62*	< 0.001
H-LE 180°/s PT (Nm)	253.31 ± 64.52	283.39 ± 59.99	204.09 ± 35.26	< 0.001
H-RF180°/s PT (Nm)	101.62 ± 33.56	117.94 ± 30.64	74.91 ± 17.11	< 0.001
H-RE 180°/s PT (Nm)	262.83 ± 68.23	299.33 ± 57.02	203.09 ± 34.4	< 0.001
15 m Time (s)	6.80 ± 0.68	6.45 ± 0.39	7.41 ± 0.66	< 0.001
Turn Time (s)	12.13 ± 0.94	11.60 ± 0.60	13.05 ± 0.67	< 0.001
100 m Time (s)	60.07 ± 4.41	57.65 ± 2.09	64.12 ± 4.43	< 0.001

S, Shoulder; H, Hip; PT, Peak Torque; F, flexor; E, extensor; L, left; R, right; IR, Internal rotation; ER, External rotation. *: p<0.05, highlighting differences in angular velocities.

**Table 3** presents the between-session reliability of all isokinetic strength tests. Intraclass correlation coefficients (ICC) ranged from 0.988 to 0.998. Coefficients of variation (CV) ranged from 1.85% to 6.25%, with left shoulder external rotation at 180°/s exhibiting a CV of 6.25%. Standard errors of measurement (SEM) ranged from 0.906 Nm to 7.941 Nm.

**Table 3:** Accompanying reliability data for each test.

Test	Total (n =32)	CV (%)(95%CI)	ICC (95%CI)	SEM
S-LF 60°/s PT (Nm)	50.41 ± 12.97	3.91 (3.30 - 4.77)	0.992 (0.986, 0.996)	1.855
S-LE 60°/s PT (Nm)	79.79 ± 22.20	2.94 (2.48 - 3.58)	0.996 (0.993 - 0.998)	2.268
S-RF 60°/s PT (Nm)	47.03 ± 12.21	4.26 (3.60 - 5.19)	0.991 (0.982 - 0.995)	1.927
S-RE 60°/s PT (Nm)	80.21 ± 22.12	2.23 (1.88 - 2.72)	0.998 (0.996 - 0.999)	1.757
S-LF180°/s PT (Nm)	48.07 ± 14.31	3.59 (3.03 - 4.37)	0.995 (0.992 - 0.998)	1.658
S-LE 180°/s PT (Nm)	78.76 ± 24.89	2.71 (2.29 - 3.30)	0.998 (0.996 - 0.999)	2.071
S-RF180°/s PT (Nm)	46.27 ± 14.07	3.58 (3.02 - 4.37)	0.995 (0.991 - 0.998)	1.589
S-RE 180°/s PT (Nm)	83.59 ± 23.82	3.12 (2.63 - 3.80)	0.996 (0.993 - 0.998)	2.513
S-LIR 60°/s PT (Nm)	44.41 ± 12.95	2.54 (2.14 - 3.10)	0.998 (0.995 - 0.999)	1.100
S-LER 60°/s PT (Nm)	28.59 ± 7.01	3.27 (2.76 - 3.99)	0.994 (0.989 - 0.997)	0.906
S-RIR 60°/s PT (Nm)	47.76 ± 14.00	3.59 (3.03 - 4.38)	0.995 (0.990 - 0.997)	1.652
S-RER 60°/s PT (Nm)	30.86 ± 7.63	3.81 (3.21 - 4.64)	0.992 (0.985 - 0.996)	1.125
S-LIR180°/s PT (Nm)	47.59 ± 15.71	3.46 (2.92 - 4.22)	0.996 (0.993 - 0.998)	1.581
S-LER 180°/s PT (Nm)	24.55 ± 8.05	6.25 (5.28 - 7.62)	0.988 (0.978 - 0.994)	1.448
S-RIR180°/s PT (Nm)	47.93 ± 14.58	2.71 (2.29 - 3.30)	0.997 (0.995 - 0.999)	1.267
S-RER 180°/s PT (Nm)	28.31 ± 10.18	3.76 (3.17 - 4.58)	0.996 (0.993 - 0.998)	1.017
H-LF 60°/s PT (Nm)	107.17 ± 24.20	3.39 (2.86 - 4.13)	0.992 (0.986 - 0.996)	3.533
H-LE 60°/s PT (Nm)	267.17 ± 59.47	3.02 (2.55 - 3.68)	0.994 (0.990 - 0.997)	7.941
H-RF 60°/s PT (Nm)	109.62 ± 30.39	3.85 (3.25 - 4.69)	0.993 (0.987 - 0.997)	4.024
H-RE 60°/s PT (Nm)	272.24 ± 74.78	2.17 (1.83 - 2.65)	0.998 (0.996 - 0.999)	5.823
H-LF180°/s PT (Nm)	104.72 ± 30.55	2.63 (2.22 - 3.21)	0.998 (0.995 - 0.999)	2.648
H-LE 180°/s PT (Nm)	253.31 ± 64.52	2.81 (2.37 - 3.42)	0.995 (0.991 - 0.998)	7.004
H-RF180°/s PT (Nm)	101.62 ± 33.56	3.96 (3.34 - 4.82)	0.995 (0.991 - 0.998)	3.837
H-RE 180°/s PT (Nm)	262.83 ± 68.23	1.85 (1.56 - 2.25)	0.998 (0.997 - 0.999)	4.732

S, Shoulder; H, Hip; PT, Peak Torque; F, flexor; E, extensor; L, left; R, right; IR, Internal rotation; ER, External rotation; CV, coefficient of variation; ICC, intraclass correlation coefficient; CI, confidence interval; SEM, standard error of measurement.

**Table 4** displays the asymmetry indices for shoulder and hip strength. For most joint actions, mean asymmetry indices were higher under the 180°/s condition than under the 60°/s condition. Mann–Whitney U tests revealed a significant sex difference in hip extension asymmetry at 180°/s, with female swimmers exhibiting greater asymmetry than male swimmers (p = 0.019). No other significant sex differences were observed for any other joint movements (all p > 0.05).

**Table 4:** Descriptive statistics of isokinetic strength asymmetry indices for shoulder and hip joints in male and female swimmers. (M ± SD).

Joint & Action	Angular Velocity	Total (n =32)	Male (n=20)	Female (n=12)	*P* (sex)
Shoulder					
Flexion (F)	60°/s	11.70 ± 6.52	10.01 ± 5.97	14.46 ± 6.69	0.092
Flexion (F)	180°/s	16.34 ± 10.69	13.67 ± 9.84	20.72 ± 11.02	0.101
Extension (E)	60°/s	8.71 ± 5.84	8.44 ± 5.40	9.15 ± 6.77	0.875
Extension (E)	180°/s	12.69 ± 8.25	10.91 ± 7.57	15.60 ± 8.83	0.2
Internal Rotation (IR)	60°/s	10.60 ± 8.41	12.04 ± 9.87	8.24 ± 4.73	0.544
Internal Rotation (IR)	180°/s	12.29 ± 9.29	11.54 ± 9.60	13.53 ± 9.06	0.457
External Rotation (ER)	60°/s	10.86 ± 8.59	12.42 ± 9.26	8.32 ± 7.00	0.225
External Rotation (ER)	180°/s	14.72 ± 9.68	16.38 ± 10.62	12.00 ± 7.58	0.558
Hip					
Flexion (F)	60°/s	10.28 ± 7.22	9.49 ± 7.04	11.57 ± 7.67	0.559
Flexion (F)	180°/s	12.37 ± 9.18	10.47 ± 6.81	15.50 ± 11.82	0.381
Extension (E)	60°/s	8.34 ± 5.53	7.56 ± 5.53	9.63 ± 5.55	0.312
Extension (E)	180°/s	8.30 ± 6.20	6.80 ± 6.47	10.75 ± 5.06	0.019*

IA, Asymmetry Index. Values in bold represent asymmetry indices at the higher angular velocity (180°/s), highlighting the general trend of greater asymmetry during faster contractions.

P values were derived from Mann–Whitney U tests for sex comparisons.

Wilcoxon signed-rank tests examined the effect of contraction velocity on asymmetry magnitude (**Table 5**). In male swimmers, the asymmetry index for shoulder external rotation was significantly higher at 180°/s than at 60°/s (Z = -2.373, p = 0.016, r = 0.531). In female swimmers, no significant velocity-dependent differences were observed for any joint action (all p > 0.05).

**Table 5:** Results of Wilcoxon signed-rank tests comparing strength asymmetry indices between slow 60°/s and fast 180°/s contraction velocities, by sex. (M ± SD).

Sex	Joint & action	60°/s IA (%)	180°/s IA (%)	MD	Z	p	r
Male (n=20)	S-F	10.01 ± 5.97	13.67 ± 9.84	+3.66	-1.677	0.099	0.375
	S-E	8.44 ± 5.40	10.91 ± 7.57	+2.47	-1.111	0.284	0.248
	S-IR	12.04 ± 9.87	11.54 ± 9.60	-0.50	-0.24	0.832	0.054
	S-ER	12.42 ± 9.26	16.38 ± 10.62	+3.96	-2.373	0.016*	0.531
	H-F	9.49 ± 7.04	10.47 ± 6.81	+0.98	-0.98	0.347	0.219
	H-E	7.56 ± 5.53	6.80 ± 6.47	-0.76	0.686	0.492	0.153
Female (n=12)	S-F	14.46 ± 6.69	20.72 ± 11.02	+6.26	-1.6	0.123	0.462
	S-E	9.15 ± 6.77	15.60 ± 8.83	+6.45	-1.689	0.102	0.488
	S-IR	8.24 ± 4.73	13.53 ± 9.06	+5.29	-0.153	0.922	0.044
	S-ER	8.32 ± 7.00	12.00 ± 7.58	+3.68	0	1	0
	H-F	11.57 ± 7.67	15.50 ± 11.82	+3.93	-1.511	0.147	0.436
	H-E	9.63 ± 5.55	10.75 ± 5.06	+1.12	-1.156	0.278	0.334

S, Shoulder; H, Hip; PT, Peak Torque; F, flexor; E, extensor; L, left; R, right; IR, Internal rotation; ER, External rotation; IA, Asymmetry Index. Mean Difference = 180°/s value - 60°/s value;

Z values are from Wilcoxon signed-rank tests. Effect sizes (r) were calculated as r = |Z|/√N, where N is the number of participants in each sex group (male: n = 20; female: n = 12), and interpreted as small (0.1), medium (0.3), or large (0.5) (Cohen, 1988). Bold values indicate significant differences between 60°/s and 180°/s asymmetry indices (p < 0.05). The bold row (S-ER for males) shows a significant velocity-dependent increase.

**Table 6** presents Spearman rank correlation coefficients between absolute strength and swimming performance. In male swimmers, significant negative correlations were observed between left shoulder extension at 60°/s and turn time (rho = -0.470, p < 0.05), and between left shoulder extension at 180°/s and turn time (rho = -0.531, p < 0.05). No other strength measures demonstrated significant correlations with performance in male swimmers. In female swimmers, significant positive correlations were observed between left hip extension at 60°/s and turn time (rho = 0.679, p < 0.05), and between right hip extension at 60°/s and turn time (rho = 0.618, p < 0.05). No other strength measures demonstrated significant correlations with performance in female swimmers.

**Table 6:** Correlation coefficients between isokinetic strength for shoulder and hip joints and performance.

°/s	PT (Nm)	15m		Turn-(45-65m)		100 m	
		Male (n=20)	Female (n=12)	Male (n=20)	Female (n=12)	Male (n=20)	Female (n=12)
60	S-LF	-0.346	0.534	-0.267	0.533	0.039	0.519
	S-LE	-0.228	0.432	-0.470*	0.301	-0.068	0.282
	S-RF	-0.247	-0.075	-0.397	0.047	-0.26	-0.14
	S-RE	-0.182	0.289	-0.348	0.237	-0.119	0.167
180	S-LF	-0.141	0.433	-0.301	0.336	0.226	0.355
	S-LE	0.018	0.528	-0.531*	0.236	-0.013	0.355
	S-RF	-0.111	-0.378	-0.365	-0.123	-0.074	-0.429
	S-RE	-0.108	0.347	-0.219	0.246	0.069	0.182
60	S-LER	-0.107	-0.099	-0.076	0.175	0.156	-0.088
	S-LIR	-0.096	0.602	-0.229	0.425	0	0.53
	S-RER	-0.21	-0.097	-0.358	0.276	-0.139	-0.037
	S-RIR	-0.085	0.374	-0.325	0.244	0.135	0.147
180	S-LER	-0.158	0.146	-0.193	0.37	0.142	0.176
	S-LIR	0.209	0.58	-0.08	0.378	0.179	0.562
	S-RER	-0.367	-0.189	-0.387	0.182	-0.19	-0.089
	S-RIR	-0.231	0.371	-0.259	0.313	0.218	0.331
60	H-LF	-0.008	0.273	-0.022	0.418	-0.079	0.255
	H-LE	-0.201	0.087	-0.486*	0.679*	0.058	0.424
	H-RF	0.179	-0.023	-0.034	0.355	0.026	-0.027
	H-RE	-0.11	0.323	-0.432	0.618*	-0.125	0.4
180	H-LF	0.192	0.274	0.163	0.269	0.252	0.141
	H-LE	-0.082	-0.132	-0.233	0.2	0.145	0.045
	H-RF	0.255	0.167	0.154	0.306	0.232	0.064
	H-RE	0.146	0.159	-0.178	0.055	0.044	-0.018

S, Shoulder; H, Hip; PT, Peak Torque; F, flexor; E, extensor; L, left; R, right; IR, Internal rotation; ER, External rotation. *p < 0.05; **p < 0.01.

**Table 7** presents Spearman rank correlation coefficients between strength asymmetry indices and swimming performance. No significant correlations were observed between any asymmetry index and start, turn, or overall 100-m time in either male or female swimmers (all p > 0.05).

**Table 7:** Correlation coefficients between isokinetic strength Asymmetry Index for shoulder and hip joints and performance.

°/s	IA (%)	15m		Turn-(45-65m)		100 m	
		Male (n=20)	Female (n=12)	Male (n=20)	Female (n=12)	Male (n=20)	Female (n=12)
60	S-F	0.04	0.314	0.127	0.173	-0.092	0.391
	S-E	0.292	0.137	-0.068	0.173	-0.451	0.236
180	S-F	0.248	-0.2	0.191	-0.255	0.375	-0.073
	SE	-0.076	-0.023	0.038	0.018	0.23	0.045
60	S-ER	0.199	-0.124	-0.012	-0.284	0.064	-0.257
	S-IR	0.151	-0.246	0.203	0.073	0.416	-0.191
180	S-ER	0.281	-0.525	0.467	-0.101	0.125	-0.43
	S-IR	-0.334	-0.132	-0.404	-0.273	0.096	-0.21
60	H-F	0.342	0.036	0.388	-0.145	0.246	0.182
	H-E	0.337	0.314	0.316	0.245	-0.171	0.164
180	H-F	0.095	0.279	0.185	0.005	0.354	0.282
	H-E	0.335	-0.241	0.038	-0.264	-0.437	-0.336

S, Shoulder; H, Hip; PT, Peak Torque; F, flexor; E, extensor; L, left; R, right; IR, Internal rotation; ER, External rotation; IA, Asymmetry Index. *p < 0.05; **p < 0.01.

## Discussion

The present study examined the characteristics of isokinetic strength asymmetry in the shoulder and hip joints of competitive swimmers and its association with sprint performance. The main findings can be summarized as follows: (1) limb asymmetry patterns exhibited movement- and velocity-specificity, with sex-dependent effects observed under high-velocity conditions; (2) in female swimmers, hip extensor strength showed significant positive correlations with turn time; and (3) strength asymmetry was generally unrelated to swimming performance, with no significant correlations observed in either male or female swimmers.

Despite pronounced sex differences in absolute strength, Mann-Whitney U tests revealed a significant sex difference in hip extension asymmetry at 180°/s, with female swimmers exhibiting greater asymmetry than male swimmers (p = 0.019). No other significant sex differences were observed for any other joint movements. This finding contrasts with studies in team sports characterized by unilateral dominance and frequent directional changes, where sex differences in asymmetry are often reported (Bishop et al., 2018; Jiang et al., 2023; Kabaciński et al., 2025; Peng et al., 2025). The discrepancy underscores the sport-specific nature of asymmetry. Swimming, as a bilateral cyclical sport, demands symmetrical force production to maintain body alignment and minimize drag (Evershed et al., 2014). Prolonged exposure to this biomechanical constraint may drive both male and female swimmers toward a convergently balanced inter-limb coordination pattern, irrespective of sex. The observed greater hip extension asymmetry in female swimmers at high velocity may reflect sex-specific differences in neuromuscular control or bilateral strength development, warranting further investigation.

A consistent trend was observed across most joint actions: asymmetry indices were higher under high-velocity (180°/s) than low-velocity (60°/s) conditions. However, velocity-dependent increases reached statistical significance only for shoulder external rotation in male swimmers (Z = -2.373, p = 0.016, r = 0.531). In female swimmers, no significant velocity-dependent differences were observed for any joint action. These findings align with the view that asymmetry may, in certain contexts, reflect individualized neuromuscular adaptation rather than functional deficit (Bishop et al., 2021). In male swimmers, heightened asymmetry in high-velocity external rotation may relate to the eccentric demands placed on the posterior rotator cuff during the rapid recovery and entry phases of front crawl, where inter-individual variation in neuromuscular control is amplified under sport-relevant speeds. The absence of significant velocity-dependent asymmetry in female swimmers suggests that their asymmetry patterns may be less influenced by contraction velocity, potentially due to differences in training history or neuromuscular adaptation. Collectively, these results suggest that compensatory training should be precisely targeted—informed by the athlete’s sex, the specific movement exhibiting asymmetry, and the contraction velocity at which it emerges—rather than universally pursuing symmetry across all joints and conditions.

A stark sex difference emerged in the association between absolute strength and swimming performance. Spearman rank correlation analysis revealed that, in female swimmers, hip extension peak torque at 60°/s showed significant positive correlations with turn time (left: rho = 0.679, p < 0.05; right: rho = 0.618, p < 0.05). This unexpected finding—that greater hip extensor strength was associated with slower turn performance—may reflect differences in swimming technique or turn strategy between male and female swimmers or may be influenced by the relatively small sample size. In contrast, no significant strength–performance correlations were observed in male swimmers, with the exception of negative correlations between left shoulder extension and turn time (60°/s: rho = -0.470, p < 0.05; 180°/s: rho = -0.531, p < 0.05). This dissociation may reflect a shift in performance-determining factors as athletes reach higher competitive levels. The male swimmers in this sample exhibited substantially greater absolute strength than their female counterparts (**Table 2**). According to the theory of diminishing returns, once a threshold of strength is attained, further gains yield progressively smaller performance benefits, and other factors—such as technical efficiency, anaerobic capacity, or pacing strategy—may become more salient (Zamparo et al., 2012; Ruiz-Navarro et al., 2025). Similar phenomena have been documented in endurance running, where economy supersedes maximal oxygen uptake as the primary differentiator among elite performers (Barnes and Kilding, 2015). Thus, for high-level male swimmers, training emphasis may need to shift from strength acquisition per se to the efficient transduction of strength into propulsive force via refined technique (Wirth et al., 2022).

Across the full test battery, strength asymmetry indices were largely unrelated to swimming performance. Spearman rank correlation analysis confirmed no significant correlations between any asymmetry index and start, turn, or overall 100-m time in either male or female swimmers (all p > 0.05). This finding is consistent with the broader literature on asymmetry in swimming: of the seven studies that have directly examined this relationship, five reported no meaningful associations (Knihs et al., 2022). Several factors may account for this pattern. First, asymmetry metrics are inherently variable; the noise inherent in a ratio-based measure derived from two raw scores reduces statistical power to detect correlations with relatively stable outcomes such as race times (Bishop et al., 2021). Second, the human motor system possesses considerable compensatory capacity. Even in the presence of measurable unilateral strength deficits, athletes may maintain symmetrical hand force production through altered muscle activation patterns or kinematic adjustments (Evershed et al., 2014). The present findings reinforce the view that, in bilateral cyclical sports, moderate inter-limb strength differences may be integrated into an individualized but still effective overall motor solution, rather than constituting a performance-limiting constraint (Maloney, 2019; D’Hondt et al., 2024). Notably, the previously reported negative correlation between hip extensor asymmetry and 100-m time in male swimmers was not replicated in the revised analysis.

Several limitations should be acknowledged. First, the sample size, particularly the female subgroup (n = 12)-was relatively modest, which may have limited statistical power and the precision of correlation estimates. Nevertheless, recruiting competitive swimmers presents inherent challenges due to limited accessibility to this population and the high cost of isokinetic testing, and our sample size is comparable to or larger than previous studies employing similar methodologies (Khiyami et al., 2022; Karpiński et al., 2020; Weston et al., 2015). Second, the cross-sectional design precludes causal inference, and the non-simultaneous nature of strength and performance testing (conducted on separate days, with intervals up to five days) may have introduced additional variability. Additionally, limb dominance was not assessed. Although **Table 2** presents left and right peak torque values separately, future studies should systematically record limb dominance to facilitate interpretation of bilateral strength data. Furthermore, asymmetry was assessed exclusively via isokinetic dynamometry in non-specific, dry-land conditions; the extent to which these measures translate to inter-limb force differences during actual swimming remains unclear. Future research should recruit larger and more diverse samples spanning multiple strokes and competitive levels, integrate dry-land strength testing with in-water kinetic and kinematic measurements to elucidate the mechanisms by which strength asymmetry does-or does not-translate into performance.

## Conclusion

In competitive swimmers, inter-limb strength asymmetry is movement-, velocity-, and sex-specific, with female swimmers exhibiting greater hip extension asymmetry at 180°/s than males. Absolute hip extensor strength is significantly associated with turn performance in female but not male swimmers, whereas shoulder extensor strength correlates negatively with turn time in males. Strength asymmetry is generally unrelated to swimming performance, with no significant correlations observed in either sex. These findings advance the theoretical understanding of limb asymmetry in cyclical sports and provide evidence-based guidance for individualized strength diagnostics and training prescription in competitive swimming. In summary, this study reveals that inter-limb strength asymmetry in a cyclical sport like swimming may represent a functional, sport-specific adaptation rather than a universal deficit.

## Data Availability

The raw data supporting the conclusions of this article will be made available by the authors, without undue reservation.
